# Assessing the impact of antimicrobial resistance policies on antibiotic use and antimicrobial resistance-associated mortality in children and adults in low and middle-income countries: a global analysis

**DOI:** 10.1136/bmjph-2023-000511

**Published:** 2025-02-11

**Authors:** Kyaw Zay Ya, Jay Patel, Günther Fink

**Affiliations:** 1 Department of Epidemiology and Public Health, Swiss Tropical and Public Health Institute, Allschwil, Switzerland; 2 University of Basel, Basel, Switzerland; 3 Centre for Population Health Sciences, The University of Edinburgh Usher Institute, Edinburgh, UK

**Keywords:** Public Health, Population Surveillance, Drug Monitoring

## Abstract

**Introduction:**

Antimicrobial resistance (AMR) poses a major threat to global health security today. In recent years, many low and middle-income countries (LMICs) have implemented policies to optimise antibiotic use in both formal and informal healthcare settings. However, there is limited evidence on the effectiveness of these national efforts in LMICs.

**Methods:**

We investigated the empirical relationship between national policies aimed at restricting antibiotic use and actual antibiotic consumption in 138 LMICs. Data on national policies were obtained from the Tripartite AMR Country Self-Assessment Survey (TrACSS) as well as from the Global Survey of Experts on AMR (GSEAR). Seven independent variables relating to AMR policies were evaluated. Outcomes included the proportion of children receiving antibiotics for lower respiratory tract infections and diarrhoea (specific to paediatric populations), along with total antibiotic consumption and AMR-associated mortality in general populations.

**Results:**

Our analysis of 138 LMICs found wide variation in antibiotic use between countries and regions. We observed strong evidence of negative association (mean difference MD=−0.150, 95% CI (−0.2593 to –0.0407)) between the presence of regulatory or legislative policies that ban over-the-counter sales of antibiotics and the proportion of children receiving antibiotic drugs for lower respiratory tract infection. Furthermore, stronger AMR governance was associated with reduced total antibiotic consumption at the country level (MD=−1.259, 95% CI (−2.297 to –0.2216)). No associations were found between other policy variables and antibiotic use or mortality.

**Conclusion:**

The results presented here suggest that there is some evidence of an empirical relationship between national policies aimed at limiting over-the-counter antibiotic sales and actual antibiotic usage practices. Further policy effectiveness research will be needed to better understand the true impact of government measures. In general, a multifaceted approach will likely be needed to fight AMR and preserve antibiotics’ effectiveness, including evidence-based policies, targeted education and research.

**WHAT IS ALREADY KNOWN ON THIS TOPIC**

- Antimicrobial resistance (AMR) poses a major threat to global health security today.
- Most of the existing evidence suggests high (and often excessive) consumption of antibiotics in both humans and animals as primary driver of AMR. At the global scale, clinically-indicated antibiotic use accounts for the majority of current antibiotic consumption.
- The Tripartite Antimicrobial Resistance Country Self-Assessment Survey (TrACSS), coordinated by the World Health Organisation (WHO), the World Organisation for Animal Health (WOAH, formerly Office International des Epizooties (OIE)), and the Food and Agriculture Organisation, served as a first global database to measure and document national responses to AMR. The more recent Global Survey of Experts on AMR (GSEAR) complements TrACSS, which is jointly administered by the Tripartite (now Quadripartite) and WHO.
- Published National Action Plans (NAPs) by the WHO Library provide further evidence of national responses to AMR.
- A recent study also examined NAPs on AMR in 114 countries. Beyond this, evidence regarding the effectiveness of national antibiotic use policies remains scarce.

**WHAT THIS STUDY ADDS**

- This study comprehensively assesses the effectiveness of current national policies aimed at restricting antibiotic use across multiple countries, analysing antibiotic usage in both paediatric and adult populations. The research incorporates the most recently available data on policies, antibiotic use and health outcomes from diverse sources.
- The findings of this study suggest that regulatory or legislative policies that ban over-the-counter sales of antibiotics may serve as a tool to optimise antibiotic use in low and middle-income countries (LMICs). There is some evidence of an association between policy and antibiotic use and sales. However, our study also highlights the need for additional evidence to fully assess the true impact of government measures.

**HOW THIS STUDY MIGHT AFFECT RESEARCH, PRACTICE OR POLICY**

- The currently available evidence suggests that antibiotic usage remains high in many LMICs, and that many policies implemented in recent years have not yielded much change in antibiotic usage at the population level.
- More comprehensive and rigorously enforced legislation is likely needed to prevent further growth in global antibiotic consumption, but it should not limit access to broad-spectrum antibiotics for treating multidrug-resistant infections.
- Implementing National Action Plans and integrating immunisation and infection prevention and control efforts may be beneficial for optimising antibiotic use in LMICs. Furthermore, research is urgently needed to identify the best strategies to translate legislative efforts into sustainable behavioural change.

## Introduction

 Antimicrobial resistance (AMR) and its spread represent a major threat to global health today.^1^,^4^ High rates of consumption of antibiotics in both humans and animals are likely the primary contributory factor to AMR; the vast majority of current antibiotic use stems from clinical settings.^25^,^7^ AMR is of particular concern in low and middle-income countries (LMICs), where antimicrobials are increasingly accessible and affordable.^7 8^ Between 2000 and 2015, the global consumption of antibiotics increased from 21.1 billion to 34.8 billion defined daily doses (DDDs).^9^ The growth in consumption was almost exclusively driven by increased usage in LMICs, where antibiotic consumption increased from 11.4 billion DDDs to 24.5 billion DDDs over the same period.^9^ On average, antibiotic exposure among children under 5 years in LMICs is up to five times higher than that observed in high-income countries, with an estimated average of 24.5 (95% CI 22.6 to 26.7) antibiotic prescriptions in the first 5 years of life,^10^ many of which appear to be prescribed for self-limiting illnesses.^10^,^12^

LMICs are disproportionately affected by AMR not only due to a higher burden of infectious diseases but also due to limited health system resources that challenge health service delivery.^13^,^16^ High volume of antibiotic use in LMICs can be attributed to factors such as precarious living conditions, lack of education and widespread beliefs based on low levels of knowledge about antibiotics.^11^,^16^ In addition, many LMICs are coping with multiple health problems at the same time, often with limited resources and a health system with limited capacity.^17^,^20^ Therefore, AMR is often not seen as a priority. In resource-limited setting of LMICs, various interventions to response AMR need to be cost-effective, affordable and support economic development.^21 22^

Antimicrobial stewardship programmes (ASPs) are supposed to play a crucial role in mitigating the spread of AMR and in promoting the rational use of antimicrobial agents.^22 23^ Although there is a clear global consensus on the need to address AMR and implement ASPs, countries’ commitment to these plans are not very clear,^23^ and rigorous evaluations of national efforts remain lacking.^24^

The main objective of this study is to assess the effectiveness of national efforts at the national and global level by investigating the empirical relationship between currently implemented national AMR policies and actual antibiotic consumption in children under 5 and adults as well as their association with AMR-associated mortality in the general populations in LMICs.

## Methods

### Study design

This is an observational cross-country study exploring the empirical relationship between national AMR policies and average antibiotic consumption as well as AMR-associated mortality. Our primary predictors of interest were legislation and regulations related to antibiotic use, de facto access to antibiotic without prescription at pharmacies, the presence of national treatment guidelines for providers, the existence of National Action Plans (NAP) on AMR and AMR governance.

### Data sources

We created a comprehensive dataset on antimicrobial legislation and usage in all 138 countries ranked as low or middle income by the World Bank in 2019.^25^ The World Bank’s 2019 income classification, which groups countries into low income, lower middle income, upper middle income and high income. We collectively referred to low, lower middle and upper middle income categories as ‘LMICs’.^25^ We compiled data from eight sources to maximise study generalisability and coverage. The primary outcome variables were the proportion of children under 5 receiving antibiotics for lower respiratory tract infection and diarrhoea treatment (specific to paediatric populations) as well as total antibiotic consumption and AMR-associated mortality in the general populations of each country. Data on antibiotic use were obtained from the Demographic and Health Surveys (DHS)^26^ and Multiple Indicator Cluster Surveys (MICS) micro datasets^27^ as well as from the Global Research on Antimicrobial Resistance (GRAM) Project.^28^ Data on AMR-associated mortality were obtained from the Institute for Health Metrics and Evaluation’s (IHME) Measuring Infectious Causes and Resistance Outcomes for Burden Estimation database.^29^ Total antibiotic consumption was obtained from the GRAM study and measured in DDDs per 1000 population per day (DDD/1000/day).^28^ We also analysed specific antibiotic classes commonly used in respiratory tract infections: penicillin, other beta-lactams and macrolides.^30^ AMR-associated deaths are defined by the IHME as deaths resulting from a drug-resistant infection, potentially caused by AMR.^4 29^ We computed AMR-associated mortality per 100 000 by dividing the reported total number of deaths by the population and subsequently multiplying the quotient by 100 000.

The analysis used a total of seven predictor variables, including four policy variables capturing legislation on antibiotic use obtained from Global Survey of Experts on AMR (GSEAR),^31^ two AMR policy implementation measures from Tripartite AMR Country Self-Assessment Survey (TrACSS)^32^ and national AMR governance published in Patel *et al* in 2023.^33^ To ensure comparability between variables, the Patel *et al* governance score was rescaled to a 0–1 scale by dividing the original score by 100.^33^ Table 1 shows the definition of the main predictor variables and our coding of the survey questions. As shown in table 1, we used legislative policies on antimicrobial use and the NAP on AMR as reported by TrACSS and GSEAR policy indicators as independent predictor variables.^31 32^ Our selection of predictor variables was driven by various considerations, focusing on the impact of different policy interventions related to antibiotic use and AMR. Legislative policies from TrACSS and GSEAR on specific regulations regarding the prescription and sale of antimicrobials, which are enforceable measures, aimed at reducing antibiotic misuse. In contrast, regulatory policies like prescription requirements from GSEAR address enforcement and the accessibility of antibiotics without a prescription, highlighting a significant issue in many LMICs, where antibiotics can often be obtained from pharmacies and informal outlets. National Treatment Guidelines were included for their role in enhancing prescribing practices, serving as interventions designed to influence healthcare workers’ clinical decisions. Similarly, policies aimed at reducing over-prescription focus on modifying healthcare worker behaviour to limit unnecessary antibiotic use, which is a key objective of global AMR strategies. The inclusion of NAPs from TrACSS, recognised as a reliable source of data on NAP implementation, aims to capture comprehensive national efforts, emphasising whether these plans are actively monitored and evaluated, potentially impacting antibiotic use. While NAPs, treatment guidelines and prescription requirements may not all be strictly legislative or regulatory, we use the term ‘antimicrobial legislation’ for broader inclusivity. Moreover, governance variables derived from the Patel study were included due to their critical role in shaping national responses to AMR.

**Table 1:** Main predictor variables

Domain	Definition	Source	Year of data collection
Legislative policies	Are you aware of any regulatory and legislative policies to ban the over-the counter sale of antibiotics without prescription in (Country)?	GSEAR	2021
Legislative policies	Does your country have laws or regulations on prescription and sale of antimicrobials, for human use?	TrACSS	2021
Prescription requirements	In your experience, how easy is it in general to get antibiotics without prescription at pharmacies, drugs shops or informal outlets in the country of your current residence?‘1’ if very easy, easy, somewhat easy or somewhat difficult.‘0’ if impossible.	GSEAR	2021
National treatment guidelines	Does (Country) have national treatment guidelines to improve antibiotic prescribing practices?	GSEAR	2021
Over-prescription	Does (Country) have policies to reduce over-prescription of antibiotics by health workers?	GSEAR	2021
National Action Plan	Country progress with development of a national action plan on AMR‘0’ if response is eitherA—No national AMR action plan.B—National AMR action plan under development orC—National AMR action plan developed‘1’ ifD—National AMR action plan being implemented orE—National AMR action plan being implemented and actively monitored through a monitoring and evaluation framework.	TrACSS	2021
Governance score	Mean antimicrobial resistance governance score from a systematic governance analysis of the National Action Plan on AMR	Patel 2023	2021

AMR, antimicrobial resistance; GSEAR, Global Survey of Experts on AMR; TrACSS, Tripartite AMR Country Self-Assessment Survey.

To address confounding concerns, we selected seven control variables based on the previous literature: vaccination coverage (DPT3: diphtheria, pertussis and tetanus vaccine); measles; total fertility rate; income per capita; urban population share; and prevalence of fever, cough and prevalence of diarrhoea.^1522 34^,^36^ Data on vaccination coverage (DPT3 and measles), total fertility rate, income per capita, urban population share variables were obtained from World Bank Development Indicators data (https://databank.worldbank.org/source/world-development-indicators).^37^ The prevalence of fever, cough and diarrhoea was extracted from DHS and MICS surveys.^26 27^

## Statistical analysis

The main empirical strategy was to assess the empirical relationship between AMR policies and antibiotic use and AMR-associated mortality at the country level using adjusted linear regression models. In order to be able to look at relative changes in outcomes, all outcome variables were log transformed. Descriptive statistics, including means, SD and frequencies, were computed to summarise the study variables. The global distribution of antibiotic use and AMR-associated mortality in LMICs was visualised using maps generated in the Quantum Geographic Information System (QGIS) mapping software. The explanatory variables are the variables described earlier, along with available data on antibiotic use in countries.

We employed standard linear regression models to estimate the strength, reliability and statistical significance of the results, facilitating the examination of relationships between variables and the quantification of predictor impact on outcomes. Statistical significance was defined as a p value <0.05. Multiple hypothesis testing corrections were not applied—standard Bonferroni corrections would imply a critical significance cut-off of 0.00142 with five outcomes and seven predictors, which is rather unlikely with a sample size of 138 countries.^38^ All presented results should thus be interpreted as exploratory. Stata/SE (V.16.0) was used for conducting the statistical analysis.^39^

### Missing data

Multiple imputation using the Stata/SE (V.16.0) mi impute package was used to impute missing covariates.^40^ We employed multiple imputation using the multivariate normal (MVN) model, assuming that all variables follow an MVN distribution. This method is particularly suitable for continuous variables, while categorical variables were treated as continuous during the imputation process. To ensure consistency between the imputation and the final analysis, all variables from the substantive models were included in the imputation procedure. The outcome variables comprised the proportions of children receiving antibiotics for respiratory infections or diarrhoea, total antibiotic consumption and AMR-associated mortality. Predictor variables, including policy-related factors and governance score, were treated as continuous. Other continuous covariates include prevalence of fever among children under age 5, prevalence of cough among children under age 5, prevalence of diarrhoea among children under age 5, DPT3 vaccination, measles vaccination, total fertility rate, income per capita and urban population share, which were also imputed as continuous and treated accordingly in the analysis. To balance computational efficiency and variability across datasets, we performed 20 imputations.^41^,^44^

### Patient and public involvement

There were no patients or public involvement in our research design, conduct or reporting. We hope to disseminate the results of our analysis to the public through an open-access publication.

## Results

**Figure 1:**
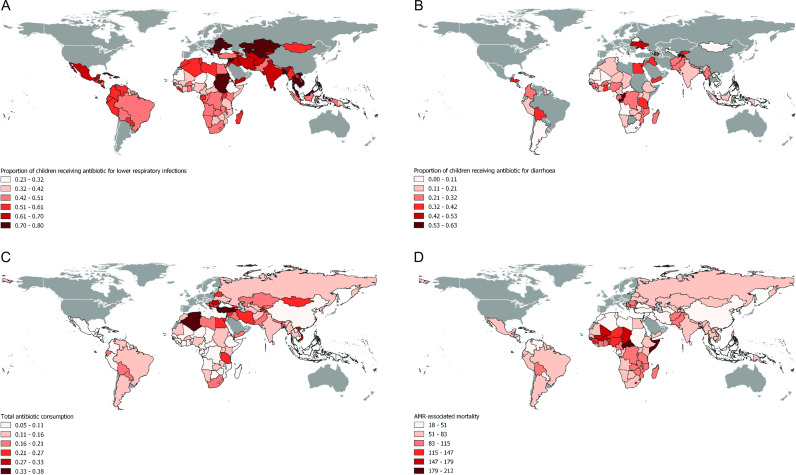
Antibiotic use, antibiotic consumption and AMR-associated mortality in LMICs. (A): Proportion of children receiving antibiotic treatment for lower respiratory infections—data from GRAM study. (B): Proportion of children receiving antibiotic treatment for diarrhoea—data from DHS and MICS. (C): Total antibiotic consumption per 1000 inhabitants per day—data from GRAM study. (D): Mortality associated with infection resistance to one or more antibiotics (deaths per 100 000 person-years)—data from IHME. Countries with available data are highlighted using a range of red shades, while those omitted due to World Bank income classification are represented in grey. Unavailable data are shown in white. AMR, antimicrobial resistance; DHS, Demographic and Health Surveys; GRAM, Global Research on Antimicrobial Resistance; IHME, Institute for Health Metrics and Evaluation; LMICs, low and middle-income countries; MICS Multiple Indicator Cluster Surveys.

Our analysis included all 138 countries classified as LMICs. For the treatment of lower respiratory infections in children, data on antibiotic use were available for 129 LMICs from the GRAM study.^4 28^ Mauritania had the lowest proportion of antibiotic use in children for respiratory infections (0.23), while Kazakhstan and Ukraine had the highest (0.80).^4 28^ Data on antibiotic use for diarrhoea treatment in children were available for 91 countries. Kiribati had the lowest antibiotic use (0.003), while Tajikistan had the highest antibiotic use for the treatment of diarrhoea (0.63).^4 28^ Total antibiotic consumption ranged between 0.05 DDD/1000 person days in the Philippines and 0.38 DDD in Tunisia.^4 28^ Somalia had the highest AMR-associated death rate (212 deaths per 100 000), while Maldives had the lowest (18 deaths per 100 000).^29^ Tunisia had the highest penicillin use (0.26 DDD/1000/day), Vietnam the highest use for betalactams (0.11 DDD/1000/day) and Montenegro the highest use for macrolides (0.052 DDD/1000/day).^28^ The lowest penicillin use was observed in the Philippines (0.01 DDD/1000/day), the lowest beta-lactams use in Burundi (0.003 DDD/1000/day) and the lowest macrolides use in the Central African Republic (0.003 DDD/1000/day).^28^ Detailed information on outcomes and predictor variables is provided in table 2. Figure 1 illustrates the proportion of antibiotic use for respiratory infections and diarrhoea in children (panels A and B) and adults (panel C) by country as well as the mortality rates associated with AMR (panel D).

**Table 2:** Indices and measures

Variables	Source	Year data collection	Variable type	LMICs with available data	Mean (SD)	Minimum	Maximum
Primary outcome variables							
Proportion of children taking antibiotic drugs for lower respiratory infections	GRAM	2018	Continuous	129	0.53 (0.14)	0.23	0.80
Proportion of children taking antibiotic drugs for diarrhoea	DHS and MICS	2005–2022	Continuous	91	0.18 (0.13)	0	0.63
Total antibiotic consumption (DDD/1000 /day)	GRAM	2018	Continuous	137	13.53 (6.90)	5	38
AMR-associated death rate including under 5	IHME	2019	Continuous	136	71.33 (31.49)	18.27	211.73
Secondary outcome variables							
Proportion of children with respiratory infections getting penicillin	GRAM	2018	Continuous	137	0.05 (0.03)	0.01	0.26
Proportion of children with respiratory infections getting beta-lactams	GRAM	2018	Continuous	137	0.02 (0.01)	0	0.10
Proportion of children with respiratory infections getting macrolides	GRAM	2018	Continuous	137	0.01 (0.01)	0	0.05
Predictor variables							
Regulatory and legislative policies to ban the over-the-counter sale of antibiotics without prescription	GSEAR	2021	Categorical	104	0.70 (0.45)	0	1
Laws or regulations on prescription and sale of antimicrobials	TrACSS	2021	Categorical	104	0.88 (0.33)	0	1
De facto access to antibiotic without prescription	GSEAR	2021	Categorical	115	0.73 (0.44)	0	1
National treatment guidelines to improve antibiotic prescribing practices	GSEAR	2021	Categorical	106	0.74 (0.44)	0	1
Policies to reduce over-prescription of antibiotics by health workers	GSEAR	2021	Categorical	108	0.64 (0.48)	0	1
National Action Plan on AMR	TrACSS	2021	Categorical	106	0.88 (0.32)	0	1
Governance score (normalised 0–1)	Patel 2023	2020–2021	Continuous	67	0.46 (0.10)	0.28	0.73
Confounders variables							
Prevalence of fever among children under age 5	DHS and MICS	2006–2022	Continuous	86	0.20 (0.07)	0.40	0.05
Prevalence of cough among children under age 5	DHS and MICS	2006–2022	Continuous	86	0.24 (0.10)	0.05	0.56
Prevalence of diarrhoea among children under age 5	DHS and MICS	2005–2022	Continuous	92	0.13 (0.06)	0.03	0.31
DPT3 vaccination	WBDI	2019	Continuous	71	75.63 (16.83)	29.80	99.00
Measles vaccination	WBDI	2019	Continuous	70	75.15 (13.67)	19.20	97.80
Total fertility rate	WBDI	2019	Continuous	131	3.11 (1.25)	1.23	6.82
Income per capita	WBDI	2019	Continuous	127	9690.19 (7127.66)	783.16	30 067.74
Urban population share	WBDI	2019	Continuous	133	51.73 (20.64)	13.25	91.99

DHS, Demographic and Health Surveys; DPT3, Diphtheria, pertussis (whooping cough), and tetanus vaccine; GRAM, Global Research on Antimicrobial Resistance; GSEAR, Global Survey of Experts on AMR; LMICs, low and middle-income countries; MICS, Multiple Indicator Cluster Surveys; TrACSS, Tripartite AMR Country Self-Assessment Survey; WBDI, World Bank Development Indicator.

Legislative policies to ban over-the-counter sale of antibiotics without prescription were reported in 70% of LMICs in the GSEAR.^31^ According to TrACSS, 88% of LMICs had policies in place to reduce the prescription and sale of antimicrobials.^32^ 64% of countries had policies aimed at reducing the overprescription of antibiotics by healthcare workers.^31^ Access to antibiotics without a prescription was possible in 74% of LMICs.^31^ National treatment guidelines for antibiotic use were observed in 74% of LMICs.^31^ 88% of LMICs had an NAP on AMR as of May 2021 (TrACSS).^32^ AMR governance scores ranged between 28 in Micronesia and 73 in Malaysia.^33^ All covariates are continuous variables in this study with the following missing data: prevalence of fever (52 missing, 37.7%), prevalence of cough (52 missing, 37.7%), prevalence of diarrhoea (46 missing, 33.3%), DPT3 vaccination (67 missing, 48.6%), measles vaccination (68 missing, 49.3%), total fertility rate (seven missing, 5.1%), income per capita (11 missing, 8.0%) and urban population share (five missing, 3.6%). Table 2 provides information on the control variables, sources of data and year of data collection. Online supplemental tables 1 and 2 provide information on the availability of data for outcome variables in each of the 138 countries examined in this study. The governance of AMR, ranked by aggregate scores on the three governance areas by country, 2020–2021, is shown in online supplemental table 3).

When examining the association between outcome variables, strong evidence of positive association was identified between total antibiotic consumption and antibiotic use for treating respiratory infections in children (coefficient=0.4064, p value <0.01). Strong evidence of negative association was observed between antibiotic use for fever in children and the AMR-associated mortality (coefficient=−0.4713, p value <0.01), while a negative association was also found between total antibiotic consumption and the AMR-associated mortality (coefficient=−0.3080, p value <0.01). Lower AMR-associated mortality is associated with higher antibiotic use for fever in children and total antibiotic use. Online supplemental tables 4 and 5 display the association between the main outcome variables in this study.

Table 3 shows the results of our bivariate analysis. In the bivariate regression models after multiple imputation, a negative association (mean difference (MD): −0.112, p<0.05, 95% CI (−0.2222 to –0.0028)) was found between legislative policies to ban the over-the-counter sale and general antibiotic use among children. There was strong evidence for a reduction in antibiotic use for the treatment of diarrhoea (MD: −0.664, p<0.01, 95% CI (−1.145 to –0.1829)). Moderate evidence of a positive association was found between total antibiotic consumption and the presence of an NAP (MD: 0.277, p<0.05, 95% CI (0.0458 to 0.5085)). No statistical evidence was found to suggest an association between AMR-associated mortality and any of the seven predictor variables analysed. The bivariate association between the antibiotic policy and the outcome variables prior to multiple imputation is found in online supplemental table 6).

**Table 3:** Bivariate correlations between antibiotic policies and outcome variables (after multiple imputation)

Bivariate	Proportion of children taking antibiotic drugs for lower respiratory tract infection	Proportion of children taking antibiotic drugs for diarrhoea	Total antibiotic consumption (DDD/1000 /day)	AMR associated mortality
		(95% CI)		
Regulatory and legislative policies to ban the over-the-counter sale	−0.112**(−0.2222 to −0.0028)	−0.664***(−1.145 to −0.1829)	−0.0114(−0.2041 to 0.1813)	0.103(−0.0776 to 0.2829)
Countries with legislation on antibiotic use	0.0141(−0.1484 to 0.1767)	−0.425(−1.113 to 0.2619)	0.0631(−0.1987 to 0.3249)	0.00256(−0.2289 to 0.2341)
Policies to reduce over-prescription of antibiotics by health workers	0.000295,(−0.1030 to 0.1036)	0.0388(−0.4242 to 0.5019)	0.135(−0.0386 to 0.3089)	−0.0810(−0.2294 to 0.0674)
De facto access to antibiotic without prescription	0.00906(−0.1160 to 0.1341)	0.326(−0.1517 to 0.8027)	0.0337(−0.1574 to 0.2248)	−0.0281(−0.2004 to 0.1441)
National treatment guidelines on antibiotic use	−0.0442(−0.1583 to 0.0698)	−0.163(−0.6657 to 0.3399)	0.0220(−0.1749 to 0.2189)	0.0152(−0.1642 to 0.1947)
National Action Plan on AMR	0.0249(−0.1152 to 0.1650)	0.0652(−0.8247 to 0.9552)	0.277**(0.0458 to 0.5085)	−0.0581 (−0.2760 to 0.1599)
Governance score on AMR	−0.241(−0.9293 to 0.4466)	0.614(−2.198 to 3.427)	−0.612(−1.470 to 0.2455)	0.204(−0.5616 to 0.9688)

Bivariate correlations between different antibiotic policies and key outcome variables related to antibiotic use in children and AMR-associated mortality. Values presented in the columns are coefficients. Coefficients indicate the strength and direction of the relationship between each predictor and the outcome variable. Negative coefficients indicate a negative correlation, while positive coefficients indicate a positive correlation. Each reported coefficient is the result of a separate regression.

Column 1: Bivariate regression for individual predictor and children's antibiotic use for lower respiratory tract infection.

Column 2: Bivariate regression for individual predictor and children's antibiotic use for diarrhoea.

Column 3: Bivariate regression for individual predictor and total antibiotic consumption (DDD/1000/day).

Column 4: Bivariate regression for individual predictor and AMR-associated mortality.

***p<0.01, **p<0.05, *p<0.1.

AMR, antimicrobial resistance; DDD, defined daily doses.

Table 4 presents associations between antibiotic policies and study outcomes. After adjusting for confounding factors, strong evidence of negative association was found between regulatory policies and antibiotic use for respiratory infections (MD: −0.150, p<0.001, 95% CI (−0.2593 to –0.0407)). We also found positive associations between log-transformed total antibiotic consumption (DDD/1000/day) and NAPs (MD: 0.417, p<0.01, 95% CI (0.0640 to 0.7709)) and a negative association between governance and log-transformed total antibiotic consumption (MD: −1.259, p<0.01, 95% CI (−2.297 to –0.2216)). An estimated MD of −1.26 indicates that a one-unit increase in the governance score was associated with a reduction of 1.26 DDD/1000/day in total antibiotic consumption. Since one SD of the governance score corresponds to approximately 0.1 units, a 1 SD increase in governance was associated with a reduction of around 0.13 DDD/1000/day. In our fully adjusted model, we found positive association between log-transformed AMR-associated mortality and regulatory and legislative policies banning over-the-counter sales (MD: 0.266, p<0.001, 95% CI (0.0772 to 0.4542)).

**Table 4:** Adjusted associations between antibiotic policies and study outcomes (after multiple imputation)

	Model 1	Model 2	Model 3	Model 4	Model 5	Model 6	Model 7	Model 8
(95% CI)								
Regulatory and legislative policies to ban the over-the-counter sale	−0.174***(−0.2707 to −0.0764)	−0.150***(−0.2593 to −0.0407)	−0.317(−0.8101 to 0.1755)	−0.375(−0.9196 to 0.1693)	−0.0793(−0.2716 to 0.1131)	−0.0439(−0.2824 to 0.1946)	0.243***(.0599 to 0.4258)	0.266***(.0772 to 0.4542)
Countries with legislation on antibiotic use	−0.0298(−0.1725 to 0.1128)	0.0209(−0.1560 to 0.1978)	−0.0547(−0.7914 to 0.6821)	−0.147(−1.077 to 0.7833)	0.00906(−0.2638 to 0.2820)	0.179(−0.1945 to 0.5515)	0.0899(−0.1340 to 0.3139)	0.103(−0.1410 to 0.3477)
De facto access to antibiotic without prescription	−0.0282-(0.1296 to 0.0731)	−0.0277(−0.1366 to 0.0813)	0.214(−0.3011 to 0.7294)	0.187(−0.3300 to 0.7045)	0.0297(−0.1909 to 0.2503)	−0.0280(−0.2694 to 0.2133)	0.0728(−0.0855 to 0.2312)	0.0773(−0.0819 to 0.2364)
National treatment guidelines on antibiotic use	−0.125**(−0.2289 to −0.0202)	−0.0573(−0.1973 to 0.0827)	0.0676(−0.3815 to 0.5166)	−0.0514(−0.6483 to 0.5456)	−0.0430(−0.2395 to 0.1535)	−0.0924(−0.3782 to 0.1935)	0.0435(−0.1249 to 0.2119)	0.0540(−0.1515 to 0.2596)
National Action Plan on AMR	−0.00973(−0.1564 to 0.1369)	0.0525(−0.1057 to 0.2108)	0.293(−0.4023 to 0.9880)	0.314(−0.3778 to 1.005)	0.231*(−0.0023 to 0.4641)	0.417**(.0640 to 0.7709)	−0.0332(−0.2493 to 0.1829)	−0.0894(−0.3166 to 0.1377)
Governance score on AMR	−0.328(−1.136 to 0.4793)	−0.192(−1.182 to 0.7984)	0.505(−1.951 to 2.962)	0.588(−2.545 to 3.721)	−0.660(−1.539 to 0.2179)	−1.259**(−2.297,−0.2216)	0.00893(−0.7026 to 0.7204)	−0.202(−1.025 to 0.6198)

Adjusted correlations between antibiotic policies and study outcomes. Values presented in the columns are coefficients. Coefficients indicate the strength and direction of the relationship between each predictor and the outcome variable. Negative coefficients indicate a negative correlation, while positive coefficients indicate a positive correlation. Each reported coefficient is the result of a separate regression.

Model 1: Individual predictor and children’s antibiotic use for lower respiratory tract infection (adjusted for control variables: prevalence of fever, cough and diarrhoea among children under age 5, dpt3 and measles vaccination, total fertility rate, income per capita, urban population share).

Model 2: All predictors and children’s antibiotic use for lower respiratory tract infection (adjusted for control variables).

Model 3: Individual predictor and children’s antibiotic use for diarrhoea (adjusted for control variables).

Model 4: All predictors and children’s antibiotic use for diarrhoea (adjusted for control variables).

Model 5: Individual predictor and total antibiotic consumption (DDD/1000 /day) (adjusted for control variables).

Model 6: All predictor and total antibiotic consumption (DDD/1000 /day) (adjusted for control variables).

Model 7: Individual predictor and AMR associated death rate (death per 100 000 populations) (adjusted for control variables).

Model 8: All predictors and AMR associated death rate (death per 100 000 populations) (adjusted for control variables).

***p<0.01, **p<0.05, *p<0.1.

AMR, antimicrobial resistance; DDD, defined daily doses.

In adjusted regression model for specific antibiotic classes shown in online supplemental table 7, the presence of an NAP on AMR was associated with a slight increase in the consumption of log-transformed penicillin for respiratory infections in children (MD: 0.447, 95% CI −0.0089 to 0.9033). While the point estimate suggests a slight increase in penicillin consumption, the CI indicates uncertainty around this estimate. For other beta-lactam antibiotics, the association was stronger (MD: 0.677, 95% CI 0.0723 to 1.282).

## Discussion

This study examined the empirical relationship between current AMR policies and the use of antibiotics in both children and adults as well as AMR-associated mortality at the country level in LMICs. To the best of our knowledge, this represents the first comprehensive assessment in terms of country coverage, evaluating the effectiveness of current national policies aimed at reducing antibiotic use on a national level. The study includes the most recent data available from 2005 to 2022 on policy, actual use of antibiotics and health outcomes from a wide range of sources. We find some evidence supporting the impact of regulatory and legislative policies, as well as stronger AMR governance, in reducing antimicrobial use. Results were most positive for over-the-counter sales bans, which were on average associated with a 0.15 reduction in the natural log of antibiotic use for the treatment of respiratory infections in children, which roughly corresponds to a 15% reduction. We also found that governance is negatively associated with the overall consumption of antibiotics. More surprisingly, we find that over-the-counter sales bans are positively associated with AMR-associated mortality. While restricting antibiotic use has the potential to slow down the spread of AMR, restricted access could in theory result in higher mortality.^45^ However, most of the currently available evidence suggests that restricting antibiotic use does not compromise treatment outcomes and patient safety.^36 45 46^ Given the cross-sectional nature of our analysis, it is possible that countries could impose such measures in response to a high mortality burden, which would then explain the unexpected positive association empirically observed.

The general use of antibiotics for the treatment of diarrhoea and respiratory infections in both children and total antibiotic consumption in the general populations show significant variation across LMICs. The highest consumption of antibiotics was observed in Central and Northern Africa, yet previous literature fails to provide a comprehensive explanation for this variance globally.^9 47^ The possible explanation that may contribute to these variations includes the burden of infectious diseases, access to diagnostic tools, sanitation, the implementation of infection and prevention control programmes and vaccination coverage.^10 11 17 48^ Our study does not present any direct results on the effect of ASPs on antibiotic use; however, the currently available evidence on ASPs underscores the urgent need for their proper implementation to optimise antibiotic use and reduces the burden of AMR in LMICs.^48^,^50^ It is crucial to ensure that antibiotics are used appropriately and only when necessary to prevent the emergence and spread of AMR. According to the GSEAR and TrACSS reports, many LMICs had policies in place to regulate the overprescription and sale of antimicrobials.^31 32^ Nonetheless, non-prescription antibiotic use remains prevalent across LMICs.^8^ Despite the introduction of antimicrobial policies and national implementation measures, their effectiveness in reducing antibiotic use in both children and adults remains uncertain.^31^ However, the findings of this study demonstrate that regulatory and legislative policies aimed at restricting over-the-counter antibiotic sales, as reported by GSEAR, seem to have some potentials in reducing antibiotic consumption for respiratory infections in children. Therefore, the enforcement of existing antibiotic policies should be considered in reducing antibiotic use globally, particularly in the context of LMICs.^31^ However, it is important to note that the policy, even if reinforced, cannot be a standalone solution.^13 51^ An expansion of behavioural AMR strategies, complemented by education, awareness and belief initiatives, along with greater involvement of the social sciences, would represent a potential solution for optimising antibiotic use in LMICs.^13 51^

At the same time, limited access to board-spectrum antibiotics remains a significant challenge for many LMICs today.^52 53^ Delayed or limited access to antimicrobials may result in higher mortality rates than those caused by resistant bacteria.^54^ A thorough policy analysis at the national level, coupled with the systematic implementation of the Access, Watch and Reserve (AWaRe) classification,^55^ is essential to ensure that life-saving antibiotics are available while access to antibiotics categorised as watch and reserve remains restricted.

Current data on mortality also suggest that AMR-associated mortality is disproportionately high in Africa, with East Africa bearing the highest burden.^29^ One of the potential explanations could be the limited accessibility to broad-spectrum and generic antibiotics for treating drug-resistant infections, which might contribute to high mortality.^53 56^ Rather than focusing solely on reducing antibiotic use, promoting the rational use of antibiotics may be more effective for addressing AMR in LMICs, where some efforts are also still necessary to ensure that those in need have access to antibiotics.^7 57^

Currently available data also suggest that between 67% and 86% of LMICs have an NAP on AMR.^32^ However, our analysis found weak association between NAP availability and related outcomes, highlighting the limited effectiveness of these plans. Instead, we found that a high NAP governance score based was linked to reduced antibiotic use. It is recommended to prioritise the operationalisation of NAPs and translate them into strategic actions that are regularly monitored and reviewed for progress.

In recent years, Clinical Decision Support Systems (CDSS) tools can improve antibiotic prescribing practices and clinical outcomes.^58 59^ These tools have shown promising results even in resource-limited settings.^60^ In LMICs, it is crucial that CDSS tools should be contextually adjusted, cost-effective and sustainable.^58^,^60^ A multifaceted approach, including the enforcement of existing policies, national implementation measures and effective interventions and tools, is necessary to slow down the spread of AMR. Evidence-informed policymaking and targeted educational campaigns for healthcare providers and patients can also promote rational antibiotic use. Additional research is needed to evaluate existing interventions, develop new strategies and address emerging challenges in the fight against AMR.

### Limitations

The study has several limitations that should be acknowledged. First, the study included IHME-derived data on AMR-associated mortality, acknowledging the reliance on model estimates due to data limitations in LMICs and the associated uncertainties. In addition, the limited availability of recent data on antibiotic use, the lack of age-specific antibiotic use data and limited policy indicators may have constrained the scope of the study. The use of data from different sources and years may introduce variability, potentially affecting the reliability of the findings. Another limitation is that the predictors of antibiotic use (ie, policies on antibiotic use and national implementation measures) were collected from GSEAR and TrACSS, which may be subjective and may not fully capture the country’s situation, including current coverage of pneumococcal and rotavirus vaccine coverage. Furthermore, there is substantial variation between countries in how antibiotic policies are framed and implemented, a limitation that should be acknowledged. The use of regression analysis to identify associations between predictors and outcomes does not establish causality and should be interpreted with caution. Despite utilising seven predictors and four primary outcomes in our hypothesis testing, we refrained from conducting multiple hypothesis tests. As a consequence, the significance of individual tests no longer accurately reflects the error rate of the combined set of tests.^38^ It is also important to acknowledge this limitation in our study. The study’s focus on human health and exclusion of One Health considerations may overlook significant factors related to AMR.^61^ While the study provides valuable insights into the factors influencing antibiotic use and AMR, these limitations should be considered when interpreting the findings and designing future research in this area.

## Conclusion

Despite a multitude of policies implemented in LMICs in recent years to slow down AMR, antibiotic use remains high in many settings, and AMR-associated mortality appears to be increasing. The results presented here suggest a somewhat weak link between national policies and the actual use of antibiotics. More comprehensive and rigorously enforced legislation is likely needed to prevent further escalation of global antibiotic consumption. However, this should not translate into restrictions on broad-spectrum antibiotics necessary for treating multidrug-resistant infections in appropriate context. It is highly recommended to prioritise the operationalisation of NAPs and the integration of immunisation and IPC targets into strategic action plans. Further research on policy effectiveness may help governments to identify the best strategies to translate legislative efforts into sustainable behavioural change.

## Supplementary material

10.1136/bmjph-2023-000511 online supplemental file 1

## Data Availability

All data relevant to the study are included in the article or uploaded as supplementary information.
