# Supplementary material for: Assessing the impact of antimicrobial resistance policies on antibiotic use and antimicrobial resistance-associated mortality in children and adults in low and middle-income countries: a global analysis
Source: BMJ Public Health. 2025 Feb 11;3(1):e000511. doi: 10.1136/bmjph-2023-000511 (PMC11843486; doi:10.1136/bmjph-2023-000511)
Supplement: online supplemental file 1 [file bmjph-3-1-s001.pdf]

Supplementary materials

Table 1 (ST1): Availability of data for outcome variables				
Country	Proportion of lower respiratory infections cases getting an antibiotic	Proportion of diarrhoea cases getting an antibiotic	Total antibiotic consumption	AMR associated death
Afghanistan	Yes	Yes	Yes	Yes
Albania	Yes	Yes	Yes	Yes
Algeria	Yes	Yes	Yes	Yes
American Samoa	Yes	No	Yes	Yes
Angola	Yes	Yes	Yes	Yes
Argentina	No	Yes	Yes	Yes
Armenia	Yes	Yes	Yes	Yes
Azerbaijan	Yes	Yes	Yes	Yes
Bangladesh	Yes	Yes	Yes	Yes
Belarus	Yes	Yes	Yes	Yes
Belize	Yes	No	Yes	Yes
Benin	Yes	Yes	Yes	Yes
Bhutan	Yes	Yes	Yes	Yes
Bolivia	Yes	Yes	Yes	Yes
Bosnia and Herzegovina	Yes	No	Yes	Yes
Botswana	Yes	No	Yes	Yes
Brazil	Yes	No	Yes	Yes
Bulgaria	Yes	No	Yes	Yes
Burkina Faso	Yes	Yes	Yes	Yes
Burundi	Yes	Yes	Yes	Yes
Cabo Verde	Yes	No	Yes	Yes
Cambodia	Yes	Yes	Yes	Yes
Cameroon	Yes	Yes	Yes	Yes
Central African Republic	Yes	Yes	Yes	Yes
Chad	Yes	Yes	Yes	Yes
China	No	No	Yes	Yes
Colombia	Yes	Yes	Yes	Yes
Comoros	Yes	Yes	Yes	Yes
Congo, Dem. Rep	Yes	Yes	Yes	Yes
Congo, Rep	Yes	Yes	Yes	Yes
Costa Rica	Yes	Yes	Yes	Yes
Cuba	Yes	Yes	Yes	Yes
Côte d'Ivoire	Yes	Yes	Yes	Yes

Djibouti	Yes	No	Yes	Yes
Dominica	Yes	No	Yes	Yes
Dominican Republic	Yes	Yes	Yes	Yes
Ecuador	Yes	No	Yes	Yes
Egypt, Arab Rep	Yes	Yes	Yes	Yes
El Salvador	Yes	No	Yes	Yes
Equatorial Guinea	Yes	No	Yes	Yes
Eritrea	Yes	No	Yes	No
Eswatini	Yes	Yes	Yes	Yes
Ethiopia	Yes	Yes	Yes	Yes
Fiji	Yes	Yes	Yes	Yes
Gabon	Yes	Yes	Yes	Yes
Gambia, The	Yes	Yes	Yes	Yes
Georgia	Yes	Yes	Yes	Yes
Ghana	Yes	Yes	Yes	Yes
Grenada	Yes	No	Yes	Yes
Guatemala	Yes	Yes	Yes	Yes
Guinea	Yes	Yes	Yes	Yes
Guinea-Bissau	Yes	Yes	Yes	Yes
Guyana	Yes	Yes	Yes	Yes
Haiti	Yes	Yes	Yes	Yes
Honduras	Yes	Yes	Yes	Yes
India	Yes	Yes	Yes	Yes
Indonesia	Yes	Yes	Yes	Yes
Iran, Islamic Rep	Yes	No	Yes	Yes
Iraq	Yes	Yes	Yes	Yes
Jamaica	Yes	No	Yes	Yes
Jordan	Yes	Yes	Yes	Yes
Kazakhstan	Yes	No	Yes	Yes
Kenya	Yes	Yes	Yes	Yes
Kiribati	Yes	Yes	Yes	Yes
Korea, Dem. People's Rep	No	No	Yes	Yes
Kosovo	Yes	No	No	No
Kyrgyz Republic	Yes	Yes	Yes	Yes
Lao PDR	Yes	Yes	Yes	Yes
Lebanon	No	Yes	Yes	Yes
Lesotho	Yes	Yes	Yes	Yes
Liberia	Yes	Yes	Yes	Yes
Libya	Yes	No	Yes	Yes
Madagascar	Yes	Yes	Yes	Yes
Malawi	Yes	Yes	Yes	Yes

Malaysia	Yes	No	Yes	Yes
Maldives	No	Yes	Yes	Yes
Mali	Yes	Yes	Yes	Yes
Marshall Islands	Yes	No	Yes	Yes
Mauritania	Yes	Yes	Yes	Yes
Mauritius	No	No	Yes	Yes
Mexico	Yes	No	Yes	Yes
Micronesia, Fed. Sts	Yes	No	Yes	Yes
Moldova	Yes	Yes	Yes	Yes
Mongolia	Yes	Yes	Yes	Yes
Montenegro	Yes	No	Yes	Yes
Morocco	Yes	No	Yes	Yes
Mozambique	Yes	Yes	Yes	Yes
Myanmar	Yes	Yes	Yes	Yes
Namibia	Yes	Yes	Yes	Yes
Nauru	No	No	Yes	Yes
Nepal	Yes	Yes	Yes	Yes
Nicaragua	Yes	No	Yes	Yes
Niger	Yes	Yes	Yes	Yes
Nigeria	Yes	Yes	Yes	Yes
North Macedonia	Yes	No	Yes	Yes
Pakistan	Yes	Yes	Yes	Yes
Papua New Guinea	Yes	Yes	Yes	Yes
Paraguay	Yes	No	Yes	Yes
Peru	Yes	Yes	Yes	Yes
Philippines	Yes	Yes	Yes	Yes
Romania	Yes	No	Yes	Yes
Russian Federation	No	No	Yes	Yes
Rwanda	Yes	Yes	Yes	Yes
Samoa	Yes	Yes	Yes	Yes
Senegal	Yes	Yes	Yes	Yes
Serbia	Yes	No	Yes	Yes
Sierra Leone	Yes	Yes	Yes	Yes
Solomon Islands	Yes	No	Yes	Yes
Somalia	Yes	No	Yes	Yes
South Africa	Yes	Yes	Yes	Yes
South Sudan	Yes	No	Yes	Yes
Sri Lanka	Yes	No	Yes	Yes
St. Lucia	Yes	Yes	Yes	Yes
St. Vincent and the Grenadines	Yes	No	Yes	Yes

Sudan	Yes	No	Yes	Yes
Suriname	Yes	Yes	Yes	Yes
Syrian Arab Republic	Yes	No	Yes	Yes
São Tomé and Principe	Yes	Yes	Yes	Yes
Tajikistan	Yes	Yes	Yes	Yes
Tanzania	Yes	Yes	Yes	Yes
Thailand	Yes	No	Yes	Yes
Timor-Leste	Yes	Yes	Yes	Yes
Togo	Yes	Yes	Yes	Yes
Tonga	Yes	Yes	Yes	Yes
Tunisia	Yes	Yes	Yes	Yes
Turkey	Yes	No	Yes	Yes
Turkmenistan	Yes	No	Yes	Yes
Tuvalu	No	Yes	Yes	Yes
Uganda	Yes	Yes	Yes	Yes
Ukraine	Yes	Yes	Yes	Yes
Uzbekistan	Yes	Yes	Yes	Yes
Vanuatu	Yes	No	Yes	Yes
Venezuela, RB	Yes	No	Yes	Yes
Vietnam	Yes	Yes	Yes	Yes
West Bank and Gaza	Yes	Yes	Yes	Yes
Yemen, Rep	Yes	Yes	Yes	Yes
Zambia	Yes	Yes	Yes	Yes
Zimbabwe	Yes	Yes	Yes	Yes

Table 2 (ST2): Data for four main outcome variables

Country	Proportion of children taking antibiotic drugs for the treatment of lower respiratory tract infection	Proportion of children taking antibiotic drugs for the treatment of diarrhoea	Total antibiotic consumption (DDD/1,000/day)	AMR associated mortality
Afghanistan	0.64	0.21	0.14	90.18
Albania	0.72	0.10	0.18	45.62
Algeria	0.61	0.19	0.37	31.61
American Samoa	0.51		0.11	56.05
Angola	0.43	0.08	0.08	71.32
Argentina		0.05	0.15	78.50
Armenia	0.46	0.18	0.13	66.84
Azerbaijan	0.48	0.39	0.11	54.30
Bangladesh	0.73	0.10	0.18	60.58

Belarus	0.79	0.05	0.22	55.79
Belize	0.46		0.09	40.48
Benin	0.30	0.07	0.10	118.06
Bhutan	0.56	0.12	0.20	60.81
Bolivia	0.48	0.38	0.20	87.35
Bosnia and Herzegovina	0.79		0.18	59.77
Botswana	0.40		0.10	77.70
Brazil	0.51		0.15	65.32
Bulgaria	0.64		0.24	91.59
Burkina Faso	0.48	0.27	0.11	149.99
Burundi	0.35	0.05	0.05	98.33
Cabo Verde	0.49		0.14	58.37
Cambodia	0.77	0.04	0.06	81.30
Cameroon	0.41	0.21	0.10	94.94
Central African Republic	0.35	0.06	0.06	199.78
Chad	0.30	0.14	0.07	157.26
China			0.08	42.80
Colombia	0.53	0.22	0.10	36.08
Comoros	0.45	0.19	0.09	75.69
Congo, Dem. Rep	0.50	0.31	0.08	87.16
Congo, Rep	0.49	0.58	0.10	67.32
Costa Rica	0.67	0.04	0.09	37.44
Cuba	0.67	0.01	0.11	60.26
Côte d'Ivoire	0.35	0.13	0.10	96.54
Djibouti	0.49		0.12	102.92
Dominica	0.59		0.11	83.56
Dominican Republic	0.59	0.21	0.09	51.24
Ecuador	0.53		0.17	53.47
Egypt, Arab Rep	0.49	0.37	0.24	56.41
El Salvador	0.47		0.06	52.58
Equatorial Guinea	0.60		0.13	46.02
Eritrea	0.38		0.07	
Eswatini	0.39	0.21	0.10	97.90
Ethiopia	0.28	0.13	0.10	76.04
Fiji	0.51	0.09	0.09	63.49
Gabon	0.57	0.29	0.10	47.13
Gambia, The	0.48	0.20	0.08	78.25
Georgia	0.64	0.03	0.24	74.59
Ghana	0.48	0.33	0.08	83.20
Grenada	0.32		0.11	64.28
Guatemala	0.52	0.33	0.06	70.84
Guinea	0.39	0.23	0.10	136.24

Guinea-Bissau	0.24	0.07	0.06	94.90
Guyana	0.35	0.12	0.09	73.97
Haiti	0.58	0.11	0.07	90.23
Honduras	0.64	0.35	0.06	53.73
India	0.67	0.19	0.14	76.11
Indonesia	0.43	0.11	0.05	49.42
Iran, Islamic Rep	0.62		0.24	30.04
Iraq	0.67	0.34	0.20	31.42
Jamaica	0.57		0.07	48.47
Jordan	0.58	0.28	0.12	23.42
Kazakhstan	0.80		0.18	51.34
Kenya	0.50	0.16	0.14	70.87
Kiribati	0.50	0.00	0.07	82.48
Korea, Dem. People's Rep			0.11	63.03
Kosovo	0.44			
Kyrgyz Republic	0.70	0.37	0.19	41.46
Lao PDR	0.59	0.06	0.09	54.62
Lebanon		0.24	0.21	27.25
Lesotho	0.42	0.16	0.10	134.05
Liberia	0.52	0.14	0.07	69.31
Libya	0.58		0.21	32.15
Madagascar	0.52	0.24	0.08	85.07
Malawi	0.46	0.13	0.10	84.53
Malaysia	0.79		0.10	43.96
Maldives		0.02	0.22	18.27
Mali	0.33	0.10	0.10	150.96
Marshall Islands	0.50		0.08	62.93
Mauritania	0.23	0.05	0.11	57.45
Mauritius			0.17	60.99
Mexico	0.65		0.07	54.94
Micronesia, Fed. Sts	0.49		0.09	60.63
Moldova	0.78	0.28	0.15	105.52
Mongolia	0.57	0.06	0.26	81.48
Montenegro	0.76		0.30	50.48
Morocco	0.53		0.15	42.36
Mozambique	0.41	0.28	0.08	102.35
Myanmar	0.54	0.26	0.11	74.44
Namibia	0.50	0.19	0.11	75.28
Nauru			0.11	46.45
Nepal	0.50	0.26	0.18	81.11
Nicaragua	0.46		0.06	37.52
Niger	0.29	0.12	0.08	134.10

Nigeria	0.33	0.30	0.15	131.05
North Macedonia	0.75		0.25	60.82
Pakistan	0.50	0.30	0.21	102.19
Papua New Guinea	0.33	0.09	0.09	82.35
Paraguay	0.52		0.18	43.34
Peru	0.56	0.20	0.10	64.18
Philippines	0.42	0.18	0.05	52.43
Romania	0.74		0.29	85.40
Russian Federation			0.13	77.65
Rwanda	0.47	0.10	0.15	77.27
Samoa	0.52	0.06	0.10	55.30
Senegal	0.33	0.13	0.10	72.72
Serbia	0.60		0.23	101.87
Sierra Leone	0.54	0.26	0.07	124.48
Solomon Islands	0.45		0.07	79.87
Somalia	0.36		0.06	211.73
South Africa	0.48	0.11	0.18	66.58
South Sudan	0.41		0.07	104.09
Sri Lanka	0.74		0.15	40.43
St. Lucia	0.60	0.05	0.09	60.72
St. Vincent and the Grenadines	0.45		0.10	72.34
Sudan	0.73		0.12	45.22
Suriname	0.49	0.09	0.11	67.08
Syrian Arab Republic	0.73		0.25	37.75
São Tomé and Principe	0.56	0.26	0.12	66.03
Tajikistan	0.74	0.63	0.23	51.13
Tanzania	0.37	0.33	0.25	93.06
Thailand	0.78		0.12	62.99
Timor-Leste	0.41	0.18	0.09	51.58
Togo	0.38	0.26	0.10	77.75
Tonga	0.46	0.09	0.10	68.90
Tunisia	0.54	0.07	0.38	32.15
Turkey	0.44		0.37	38.21
Turkmenistan	0.61		0.14	48.30
Tuvalu		0.03	0.11	68.64
Uganda	0.53	0.19	0.11	69.45
Ukraine	0.80	0.49	0.13	70.92
Uzbekistan	0.78	0.09	0.13	51.22
Vanuatu	0.47		0.09	55.36
Venezuela, RB	0.54		0.16	42.07
Vietnam	0.74	0.02	0.30	54.44

West Bank and Gaza	0.70	0.37	0.15	29.15
Yemen, Rep	0.64	0.34	0.16	55.43
Zambia	0.45	0.16	0.11	87.16
Zimbabwe	0.42	0.17	0.10	107.60

Table 3 (ST3): Antimicrobial resistance governance ranked by aggregate scores on three governance areas and 18 domains by country, 2020–21Reproduced from Patel et al (The Lancet 2023) Measuring the global response to antimicrobial resistance, 2020–21: a systematic governance analysis of 114 countries																					
country	governance_score	policy_design	implementation	monitor_evaluate	strategic_vision	coordination	participation	accountability	transparency	sustainability	equity	surveillance	stewardship	ipc	education	awareness	regulation	research	reporting	feedback	effectiveness	resistance_research
Afghanistan	0.35	0.42	0.27	0.38	0.23	0.31	0.61	0.17	0.75	0.38	0.5	0.18	0.2	0.48	0.06	0.12	0.33	0.5	0.33	0.08	0.5	0.63
Argentina	0.4	0.35	0.54	0.16	0.25	0.44	0.61	0.17	0.5	0.28	0	0.66	0.83	0.65	0.25	0.19	0.67	0.33	0.38	0.17	0	0
Bangladesh	0.39	0.49	0.38	0.19	0.37	0.5	0.72	0.44	0.63	0.31	0.5	0.25	0.44	0.75	0.25	0.14	0.67	0	0.5	0	0	0.13
Bhutan	0.42	0.47	0.43	0.28	0.84	0.5	0.67	0.11	0.5	0.3	0	0.5	0.69	0.67	0.08	0.12	0.67	0	0.33	0	0.5	0.25
Brazil	0.45	0.54	0.46	0.21	0.28	0.44	0.94	0.17	0.88	0.53	0.5	0.45	0.41	0.83	0.25	0.19	0.67	0.33	0.54	0	0	0.13
Burkina Faso	0.54	0.63	0.54	0.35	0.67	0.44	1	0.17	1	0.54	0	0.48	0.61	0.75	0.47	0.51	0.67	0.17	0.63	0	0	0.63
Cambodia	0.39	0.53	0.32	0.26	0.75	0.44	0.89	0.17	0.5	0.26	1	0.32	0.37	0.52	0.36	0.1	0	0.5	0.38	0	0.5	0.13
Cameroon	0.38	0.55	0.19	0.5	0.67	0.17	0.78	0.17	0.88	0.44	0.5	0.04	0.13	0.35	0.17	0.14	0	0.5	0.5	0.25	0.5	0.75
China	0.64	0.61	0.8	0.27	0.86	1	0.83	0.33	0.5	0.46	0	0.82	0.9	0.79	0.28	0.83	0.92	1	0.42	0.33	0	0.25
Colombia	0.49	0.52	0.55	0.25	0.4	1	0.94	0.33	0.38	0.49	0	0.62	0.6	0.9	0.42	0.49	0.67	0	0.13	0.83	0	0.13
Costa Rica	0.34	0.32	0.46	0.08	0.34	0.35	0.78	0	0.25	0.19	0.5	0.52	0.64	0.65	0.39	0.17	0.67	0	0.04	0.17	0	0.13
Cuba	0.48	0.49	0.62	0.11	0.17	1	1	0.33	0.38	0.5	0	0.68	0.66	0.94	0.47	0.58	0.83	0	0.17	0.25	0	0
Ecuador	0.43	0.44	0.49	0.24	0.8	0.44	1	0	0.25	0.29	0	0.57	0.58	0.77	0.25	0.21	0.75	0.17	0.04	0.5	0.5	0
Eritrea	0.4	0.53	0.33	0.28	0.64	0.44	1	0	0.5	0.54	0.5	0.33	0.2	0.58	0.08	0.58	0.33	0.17	0	0	0.5	0.75
Eswatini	0.47	0.49	0.54	0.24	0.76	0.63	1	0.11	0.25	0.44	0	0.51	0.75	0.75	0.39	0.17	0.67	0.33	0	0.33	0.5	0.25
Ethiopia	0.51	0.52	0.6	0.25	0.35	0.77	0.89	0.39	0.5	0.42	0.5	0.7	0.73	0.88	0.53	0.56	0.67	0	0.38	0.42	0	0.13
Fiji	0.32	0.38	0.34	0.14	0.2	0.69	0.72	0.22	0.38	0.34	0	0.01	0.37	0.5	0.25	0.49	0.67	0	0	0	0.5	0.13
Georgia	0.45	0.53	0.41	0.4	0.27	0.73	0.78	0.39	0.63	0.63	0	0.49	0.4	0.52	0.33	0.46	0.33	0.33	0.33	0.67	0.5	0.13
Ghana	0.57	0.71	0.46	0.57	0.81	0.77	0.89	0.39	0.75	0.67	0.5	0.38	0.43	0.65	0.22	0.49	0.33	0.67	0.38	0	1	1
India	0.51	0.48	0.52	0.53	0.6	0.54	0.67	0.28	0.5	0.32	0.5	0.44	0.52	0.6	0.39	0.14	0.67	0.83	0.38	0.08	1	0.75
Indonesia	0.53	0.53	0.61	0.29	0.69	0.4	0.89	0.17	0.63	0.24	1	0.62	0.61	0.9	0.25	0.49	0.83	0.5	0.38	0.5	0	0.25

Iran, Islamic Rep	0.57	0.53	0.64	0.44	0.88	0.54	0.78	0.28	0.5	0.35	0	0.87	0.68	0.96	0.53	0.17	0.67	0.5	0.46	0.67	0.5	0.13
Iraq	0.48	0.69	0.37	0.27	0.59	0.92	0.89	0.5	0.75	0.68	0.5	0.49	0.48	0.73	0.08	0.14	0.5	0	0.38	0.42	0	0.25
Jordan	0.53	0.65	0.49	0.37	0.9	0.4	0.94	0.33	0.75	0.51	0.5	0.62	0.56	0.65	0.28	0.54	0.67	0	0.42	0.42	0	0.63
Kenya	0.51	0.47	0.61	0.3	0.66	0.5	0.61	0.28	0.5	0.28	0.5	0.73	0.82	0.85	0.42	0.56	0.67	0	0.38	0.17	0.5	0.13
Lao PDR	0.5	0.5	0.53	0.43	0.81	0.4	0.72	0.17	0.5	0.2	1	0.32	0.83	0.71	0.22	0.19	0.67	0.5	0.38	0	1	0.38
Lebanon	0.45	0.52	0.41	0.36	0.73	0.5	0.56	0.28	0.63	0.52	0	0.35	0.66	0.54	0.25	0.19	0.67	0	0.33	0.5	0.5	0.13
Liberia	0.56	0.62	0.57	0.38	0.75	0.44	1	0.17	0.75	0.54	0.5	0.29	0.64	0.92	0.22	0.58	0.67	0.5	0.33	0.08	0.5	0.63
Libya	0.31	0.46	0.2	0.25	0.72	0.08	0.56	0.17	0.63	0.35	0.5	0	0.26	0.35	0.28	0.1	0.33	0	0.33	0	0.5	0.13
Madagascar	0.49	0.55	0.44	0.44	0.5	0.44	1	0.17	0.75	0.54	0	0.26	0.46	0.85	0.06	0.17	0.67	0.5	0.33	0.25	0.5	0.75
Malawi	0.55	0.77	0.39	0.44	0.77	0.96	0.94	0.5	0.75	0.72	1	0.4	0.27	0.75	0.06	0.2	0.33	0.67	0.33	0.25	0.5	0.75
Malaysia	0.73	0.66	0.85	0.6	0.7	0.96	0.94	0.5	0.63	0.47	0.5	0.99	0.99	0.92	0.64	0.95	0.83	0.5	0.83	0.83	0.5	0.13
Maldives	0.33	0.5	0.24	0.17	0.52	0.65	0.72	0.39	0.5	0.32	0.5	0.01	0.15	0.44	0.06	0.17	0.33	0.5	0.33	0	0	0.25
Mauritius	0.33	0.39	0.32	0.22	0.77	0.23	0.61	0.17	0.5	0.09	0	0.26	0.35	0.56	0.19	0.1	0.67	0	0.33	0	0.5	0
Mexico	0.55	0.5	0.63	0.44	0.8	0.58	0.89	0.11	0.38	0.4	0	0.64	0.71	0.88	0.11	0.54	0.75	0.67	0.04	0.67	1	0.25
Micronesia, Fed. Sts	0.28	0.42	0.16	0.29	0.66	0.25	1	0	0.25	0.29	0.5	0.19	0.12	0.38	0.31	0.07	0	0	0	0.17	1	0.13
Mongolia	0.31	0.28	0.39	0.17	0.47	0.27	0.22	0	0.5	0.1	0.5	0.31	0.48	0.75	0.22	0.11	0.67	0	0.25	0.25	0	0.13
Montenegro	0.38	0.59	0.3	0.11	0.48	0.92	0.72	0.33	0.75	0.64	0	0.3	0.41	0.42	0.03	0.14	0.67	0	0.33	0	0	0
Morocco	0.54	0.61	0.56	0.36	0.36	0.77	0.94	0.39	0.75	0.69	0	0.51	0.87	0.58	0.56	0.26	0.92	0	0.54	0.67	0	0.13
Mozambique	0.57	0.61	0.55	0.54	0.59	0.63	1	0.44	0.5	0.44	1	0.6	0.82	0.92	0.28	0.29	0.67	0	0.42	0.67	1	0.13
Myanmar	0.53	0.62	0.49	0.44	0.6	0.81	1	0.39	0.63	0.47	0.5	0.42	0.42	0.85	0.28	0.56	0.33	0.5	0.58	0.25	0.5	0.38
Namibia	0.45	0.41	0.47	0.47	0.46	0.31	0.83	0	0.5	0.19	1	0.61	0.49	0.79	0.42	0.24	0.67	0	0.33	0.5	1	0.13
Nepal	0.43	0.45	0.49	0.21	0.28	0.54	0.83	0.28	0.63	0.36	0	0.51	0.6	0.6	0.25	0.17	0.67	0.5	0.38	0.25	0	0.13
Nicaragua	0.38	0.4	0.45	0.13	0.48	0.44	1	0	0.25	0.29	0.5	0.49	0.53	0.77	0.22	0.19	0.75	0	0.08	0.33	0	0.13
Nigeria	0.55	0.66	0.52	0.36	0.87	0.77	0.94	0.39	0.63	0.44	0.5	0.54	0.42	0.54	0.47	0.88	0.33	0.5	0.5	0.25	0.5	0.13
North Macedonia	0.46	0.47	0.56	0.19	0.64	0.54	0.72	0.28	0.5	0.33	0	0.75	0.59	0.65	0.25	0.88	0.75	0	0.5	0.08	0	0
Pakistan	0.41	0.51	0.34	0.38	0.79	0.5	0.67	0.28	0.63	0.3	0	0.42	0.52	0.58	0.19	0.1	0.33	0	0.5	0.33	0.5	0.13
Papua New Guinea	0.42	0.61	0.26	0.4	0.7	0.54	0.72	0.44	0.63	0.58	0.5	0.35	0.29	0.38	0.06	0.05	0.67	0	0	0.17	1	0.63
Paraguay	0.36	0.4	0.42	0.12	0.32	0.44	0.89	0.17	0.38	0.26	0.5	0.4	0.51	0.58	0.36	0.17	0.33	0.5	0.04	0.33	0	0.13
Peru	0.61	0.8	0.46	0.57	0.88	0.92	0.78	0.83	1	0.65	0	0.23	0.38	0.75	0.33	0.12	0.67	0.67	0.54	0	1	0.75
Philippines	0.71	0.76	0.68	0.71	0.81	0.96	0.83	0.5	0.75	0.69	1	0.64	0.79	0.85	0.28	0.54	0.67	0.83	0.38	0.75	1	0.88

Rwanda	0.45	0.53	0.48	0.17	0.64	0.44	1	0	0.5	0.54	0.5	0.17	0.38	0.94	0.44	0.31	0.67	0.33	0	0	0	0.75
Serbia	0.48	0.54	0.55	0.18	0.78	0.58	0.89	0.28	0.5	0.4	0	0.4	0.6	0.73	0.31	0.54	0.67	0.5	0	0.17	0.5	0.13
Sierra Leone	0.29	0.4	0.24	0.14	0.33	0.4	0.94	0.17	0.38	0.26	0.5	0.18	0.12	0.58	0.33	0.13	0.33	0	0	0	0.5	0.13
South Africa	0.43	0.52	0.42	0.25	0.33	0.88	0.67	0.5	0.63	0.35	0.5	0.56	0.56	0.65	0.22	0.15	0.67	0	0.5	0.25	0	0.13
Sri Lanka	0.51	0.52	0.51	0.44	0.54	0.44	0.94	0.17	0.63	0.28	1	0.57	0.59	0.73	0.25	0.17	0.67	0.5	0.5	0	1	0.25
Sudan	0.43	0.55	0.32	0.44	0.71	0.31	0.67	0.17	0.75	0.4	1	0.24	0.46	0.54	0.08	0.44	0.33	0	0.42	0.25	0.5	0.63
Tajikistan	0.38	0.57	0.35	0.03	0.49	0.96	0.89	0.5	0.38	0.45	0.5	0.22	0.41	0.54	0.25	0.26	0.67	0	0	0	0	0.13
Tanzania	0.59	0.7	0.55	0.45	0.79	0.69	0.83	0.39	0.75	0.62	1	0.59	0.8	0.71	0.31	0.56	0.67	0	0.33	0.42	0.5	0.63
Thailand	0.72	0.72	0.69	0.8	0.89	1	1	0.5	0.63	0.5	0.5	0.92	0.78	0.94	0.42	0.88	0.75	0	0.83	0.83	0.5	1
Timor-Leste	0.52	0.56	0.51	0.49	0.55	0.63	0.78	0.28	0.75	0.39	0.5	0.53	0.35	0.73	0.19	0.61	0.67	0.5	0.5	0.33	1	0.13
Tunisia	0.39	0.4	0.42	0.29	0.57	0.31	0.67	0.17	0.63	0.15	0	0.44	0.46	0.56	0.28	0.14	0.67	0.33	0.33	0.17	0.5	0.13
Turkmenistan	0.31	0.44	0.24	0.16	0.45	0.73	0.67	0.39	0.25	0.35	0.5	0.25	0.25	0.48	0.17	0.14	0.33	0	0	0.08	0.5	0.13
Uganda	0.56	0.66	0.53	0.39	0.83	0.5	0.78	0.28	0.75	0.58	1	0.61	0.62	0.56	0.25	0.14	0.67	0.83	0.33	0	0.5	0.75
Ukraine	0.29	0.3	0.39	0	0.22	0.31	0.72	0.17	0.38	0.16	0	0.27	0.5	0.77	0.11	0.17	0.67	0	0	0	0	0
Zambia	0.54	0.55	0.57	0.42	0.58	0.31	0.78	0.17	0.75	0.42	1	0.52	0.73	0.77	0.33	0.17	0.83	0.5	0.33	0.25	0.5	0.63
Zimbabwe	0.59	0.62	0.62	0.45	0.52	0.63	1	0.28	0.63	0.69	0.5	0.36	0.65	0.83	0.33	0.58	0.67	0.83	0.33	0.42	0.5	0.63

Table 4 (ST4): Correlation matrix of Policy Measures and Antibiotic Use				
Outcome	Proportion of lower respiratory infections cases getting an antibiotic	Proportion of diarrhoea cases getting an antibiotic	Total antibiotic consumption	AMR associated death
Proportion of respiratory infections getting an antibiotic	1.0000			
Sig				
Proportion of diarrhoea cases getting an antibiotic	0.1746	1.0000		
Sig	0.1057			
Total antibiotic consumption	0.4064*	0.0837	1.0000	
Sig	0.0000	0.4302		
AMR associated death	-0.4713*	-0.0668	-0.3080*	1.0000
Sig	0.0000	0.5292	0.0003	
Sig * p<0.05				

Table 5 (ST5): Correlation matrix of Policy Measures and Antibiotic Use (in Log)				
Outcome	Proportion of lower respiratory infections cases getting an antibiotic	Proportion of diarrhoea cases getting an antibiotic	Total antibiotic consumption	AMR associated death
Proportion of respiratory infections getting an antibiotic	1.0000			
Sig				
Proportion of diarrhoea cases getting an antibiotic	0.0470	1.0000		
Sig	0.6654			
Total antibiotic consumption	0.4296*	0.0641	1.0000	
Sig	0.0000	0.5462		
AMR associated death	-0.4888*	0.0307	-0.3452*	1.0000
Sig	0.0000	0.7726	0.0000	
Sig * p<0.05				

Table 6 (ST6): Bivariate correlations between antibiotic policies and outcome variables (before Multiple imputation)				
Bivariate	Proportion of children taking antibiotic drugs for the treatment of lower respiratory tract infection<br>1	Proportion of children taking antibiotic drugs for the treatment of diarrhoea<br>2	Total antibiotic consumption (DDD/1,000/day)<br>3	AMR associated mortality<br>4
		(95% CI)		
Regulatory and legislative policies to ban the over-the counter sale	-0.117**<br>(-.2309, -.0036)	-0.526***<br>(-.1517, .1950)	0.0391<br>(-.1592, .2373)	0.106<br>(-.0660, .2782)
Countries with legislation on antibiotic use	0.0216<br>(-.1517, .1950)	-0.318<br>(-.9149, .2791)	0.0991<br>(-.1896, .3877)	-0.0337<br>(-.2954, .2280)
Policies to reduce over-prescription of antibiotics by health workers	-0.0226<br>(-.1353, .0901)	-0.0145<br>(-.3909, .3619)	0.183*<br>(-.0008, .3659)	-0.0898<br>(-.2661, .0864)
De facto access to antibiotic without prescription	0.0165<br>(-.1018, .1349)	0.360<br>(-.0794, .8002)	0.0340<br>(-.1630, .2311)	-0.0273<br>(-.2053, .1508)
National treatment guidelines on antibiotic use	-0.0625<br>(.1895, .0568)	-0.228<br>(-.6374, .1804)	0.0834<br>(-.1171, .2838)	0.0293<br>(-.1570, .2156)
National Action Plan on AMR	0.0229<br>(-.1437, .1895)	-0.0323<br>(-.6300, .5654)	0.302**<br>(.0286, .5748)	-0.0849<br>(-.3368, .1670)
Governance score on AMR	-0.323<br>(-.9065,.2612)	2.260*<br>(-.2615, 4.781)	0.302**<br>(-2.279, -.1994)	0.310<br>(-.6838, 1.304)

*** p<0.01, ** p<0.05, * p<0.1, CI= Confidence Interval in parentheses

Column 1: Bivariate regression for individual predictor and children's antibiotic use for lower respiratory tract infection. Each reported coefficient is the result of a separate regression.

Column 2: Bivariate regression for individual predictor and children's antibiotic use for diarrhoea. Each reported coefficient is the result of a separate regression.

Column 3: Bivariate regression for individual predictor and total antibiotic consumption (DDD/1,000/day). Each reported coefficient is the result of a separate regression.

Column 4: Bivariate regression for individual predictor and AMR associated mortality.
Each reported coefficient is the result of a separate regression.

The table shows bivariate correlations between different antibiotic policies and key outcome variables related to antibiotic use in children and AMR-associated mortality. The values presented in the column are coefficients. Coefficients indicate the strength and direction of the relationship between each predictor and the outcome variable. Negative coefficients indicate a negative correlation, while positive coefficients indicate a positive correlation. Each reported coefficient is the result of a separate regression.

Table 7 (ST7): Adjusted associations between antibiotic policies and specific antibiotic consumption						
	Penicillin consumption Model 1	Penicillin consumption Model 2	Betalactams consumption Model 3	Betalactams consumption Model 4	Macrolides consumption Model 5	Macrolides consumption Model 6
(95% CI)						
Regulatory and legislative policies to ban the over-the counter sale	-0.173 (-.4255, .07960)	-0.160 (-.4552, .1343)	-0.334* (-.6743, .0061)	-0.267 (-.6386, .1049)	-0.0808 (-.2985, .1369)	-0.0538 (-.3059, .1983)
Countries with legislation on antibiotic use	-0.0316 (-.3795, .3163)	0.0601 (-.3560, .4763)	-0.230 (-.6663, .2060)	0.0168 (-.5851, .6188)	-0.0229 (-.2998, .2541)	0.129 (-.2652, .5236)
Policies to reduce over-prescription of antibiotics by health workers	0.124 (-.1016, .3503)	0.247* (-.0386, .5319)	0.0466 (-.2647, .3580)	0.322* (-.0690, .7128)	0.0443 (-.1404, .2290)	0.143 (-.1020, .3871)
De facto access to antibiotic without prescription	0.116 (-.1473, .3783)	0.0941 (-.1812, .3694)	-0.0997 (-.4043, .2049)	-0.122 (-.4357, .1922)	-0.0235 (-.2153, .1684)	-0.0491 (-.2550, .1567)
National treatment guidelines on antibiotic use	-0.0303 (-.2868, .2263)	-0.128 (-.4758, .2191)	-0.270* (-.5822, .0430)	-0.318 (-.7256, .0905)	-0.0679 (-.2643, .1285)	-0.0923 (-.3594, .1749)
National Action Plan on AMR	0.268 (-.0685, .6040)	0.447** (-.0089, .9033)	0.300 (-.1136, .7128)	0.677** (0723, 1.282)	0.176 (-.0824, .4350)	0.379* (-.0167, .7749)
Governance score on AMR	-0.490 (-1.855, .8742)	-1.003 (-2.879, .8724)	-1.434* (-3.032, .1640)	-1.861* (-4.173, .4514)	-0.705 (-1.597, .1875)	-1.200* (-2.496, .0959)
*** p<0.01, ** p<0.05, * p<0.1, CI=Confidence Interval in parentheses<br>Model 1: Individual predictor and penicillin consumption in DDD/1,000/day (adjusted for control variables: prevalence of fever, cough and diarrhoea among children under age 5, dpt3 and measles vaccination, total fertility rate, income per capita, urban population share)						

Model 2: All predictors and penicillin consumption in DDD/1,000/day (adjusted for control variables)
Model 3: Individual predictor and Betalactams consumption in DDD/1,000/day (adjusted for control variables)
Model 4: All predictors and Betalactams consumption in DDD/1,000/day (adjusted for control variables)
Model 5: Individual predictor and Macrolides consumption in DDD/1,000/day (adjusted for control variables)
Model 6: All predictor and Macrolides consumption in DDD/1,000/day (adjusted for control variables)
Each reported coefficient is the result of a separate regression.
The table shows adjusted correlations between antibiotic policies and specific antibiotic consumption. The values presented in the column are coefficients.
Coefficients indicate the strength and direction of the relationship between each predictor and the outcome variable. Negative coefficients indicate a negative correlation, while positive coefficients indicate a positive correlation. Each reported coefficient is the result of a separate regression.
